# In-hospital mortality and successful weaning from venoarterial extracorporeal membrane oxygenation: analysis of 5,263 patients using a national inpatient database in Japan

**DOI:** 10.1186/s13054-016-1261-1

**Published:** 2016-04-05

**Authors:** Shotaro Aso, Hiroki Matsui, Kiyohide Fushimi, Hideo Yasunaga

**Affiliations:** Department of Clinical Epidemiology and Health Economics, School of Public Health, The University of Tokyo, 7-3-1 Hongo, Bunkyo-ku, Tokyo, 113-0033 Japan; Department of Health Policy and Informatics, Tokyo Medical and Dental University Graduate School of Medicine, 1-5-45 Yushima, Bunkyo-ku, Tokyo, 113-8510 Japan

**Keywords:** Age factors, Cardiogenic shock, Extracorporeal membrane oxygenation, Cardiac arrest, Mortality

## Abstract

**Background:**

The mortality rate of severely ill patients treated with venoarterial extracorporeal membrane oxygenation (VA-ECMO) remains unknown because of differences in patient background, clinical settings, and sample sizes between studies. We determined the in-hospital mortality of VA-ECMO patients and the proportion weaned from VA-ECMO using a national inpatient database in Japan.

**Methods:**

Patients aged ≥19 years who received VA-ECMO during hospitalization for cardiogenic shock, pulmonary embolism, hypothermia, poisoning, or trauma between 1 July 2010 and 31 March 2013 were identified, using The Japanese Diagnosis Procedure Combination national inpatient database.

**Results:**

The primary outcome was in-hospital mortality and the secondary outcome was the proportion weaned from VA-ECMO. A total of 5263 patients received VA-ECMO during the study period. The majority of patients had cardiogenic shock (n = 4,658). The number of patients weaned from VA-ECMO was 3389 (64.4 %) and in-hospital mortality after weaning from VA-ECMO was 1994 (37.9 %). In-hospital mortality without cardiac arrest in the cardiogenic shock group was significantly lower than that in patients with cardiac arrest (70.5 % vs. 77.1 %, *p* <0.001). In the multivariable logistic regression including multiple imputation, higher age and greater or smaller body mass index were significantly associated with in-hospital mortality, whereas hospital volume was not associated with such mortality.

**Conclusions:**

The present nationwide study showed high mortality rates in patients who received VA-ECMO, and in particular in patients with cardiogenic shock and in patients with cardiac arrest. Weaning from VA-ECMO did not necessarily result in survival. Further studies are warranted to clarify risk-adjusted mortality of VA-ECMO using more detailed data on patient background.

## Background

Venoarterial extracorporeal membrane oxygenation (VA-ECMO) is one of the life-supporting procedures that may be given to critically ill patients with refractory cardiogenic shock, pulmonary embolism, hypothermia, or drug poisoning [1, 2]. VA-ECMO can be used temporarily in critically ill patients until intensive therapies for underlying diseases take effect. Thus, VA-ECMO is considered to be a last resort to save life.

The reported mortality of patients with refractory cardiogenic shock treated with VA-ECMO varies widely (24–64 %), and may be related to differences in patient background and clinical settings. Previous studies of VA-ECMO have also been limited because of the small sample sizes (15–202 patients) [3–14]. The Extracorporeal Life Support Organization (ELSO) Guidelines for Adult Cardiac Failure state that the estimated proportion of patients surviving to discharge after VA-ECMO for cardiogenic shock is 40 % [15]. However, there are few data on the effectiveness of VA-ECMO for diseases or conditions other than cardiogenic shock. Hypothermia is the second most frequent disorder for which VA-ECMO is used, but the sample sizes of previous hypothermia studies (n = 24–68) [16–19] were smaller than those of cardiogenic shock studies. To date, the indication for, mortality in, or successful weaning from, VA-ECMO remains unknown for diseases or conditions other than cardiogenic shock.

The aims of this study were to investigate the in-hospital mortality of patients receiving VA-ECMO and the proportions of patients weaned from VA-ECMO, using data from a national inpatient database in Japan.

## Methods

This study was approved by the Institutional Review Board of The University of Tokyo. The requirement for informed patient consent was waived because of the anonymous nature of the data.

### Data source

The Japanese Diagnosis Procedure Combination (DPC) database includes administrative claims and discharge abstract data for all inpatients discharged from more than 1000 participating hospitals nationwide. It covers approximately 92 % (244/266) of all tertiary-care emergency hospitals in Japan. The database includes the following patient information: age; sex; body weight and height; primary diagnosis; comorbidities on admission; post-admission complications classified according to the International Classification of Diseases 10th Revision (ICD-10) codes; medical procedures designated using original Japanese codes, including VA-ECMO, intra-aortic balloon pumping (IABP), pulmonary artery catheterization, and continuous renal replacement therapy; daily records of drug administration and devices used; length of stay; and discharge status. The dates of hospital admission, surgery, bedside procedures, drugs administered and hospital discharge are recorded using a uniform data submission form. This study used data from 1 July 2010 to 31 March 2013.

### Patient selection

We included patients aged ≥19 years who received VA-ECMO during hospitalization. We selected patients who were diagnosed at admission as having cardiogenic shock (ICD-10 codes, I05, I07, I08, I20-22, I33-35, I40-42, I46, and I49-51), pulmonary embolism (I26), hypothermia (T68), poisoning (T36-65), or trauma (S$ and T0). We excluded patients with acute respiratory distress syndrome.

### Variables and outcomes

Baseline variables included age, sex and body mass index (BMI). Patients with cardiogenic shock were categorized in four age groups: 19–39, 40–59, 60–79 and ≥80 years. BMI was categorized as being underweight (BMI <18.5 kg/m^2^), normal weight (18.5–24.9 kg/m^2^), or overweight (≥25.0 kg/m^2^). Patients were also divided into those who had or who had not had an episode of cardiac arrest. Hospital volume was defined as the number of patients receiving VA-ECMO per year at each hospital. Hospital volume was categorized in tertiles and so the number of patients in each category was almost equal. Myocarditis, chronic renal failure, liver failure, and central nervous system dysfunction were extracted based on ICD-10 codes. The primary outcome was in-hospital mortality. The secondary outcome was the proportion of patients weaned from VA-ECMO.

### Statistical analysis

Data are presented as percentages and numbers, or means and standard deviations. The chi-square test and Fisher’s exact test were performed to compare proportions in the different groups. In the cardiogenic shock group, we performed multivariable logistic regression analysis to examine the association of in-hospital mortality with various factors, including several components of the survival after venoarterial ECMO (SAVE) score for VA-ECMO (age, sex, weight, myocarditis (ICD-10 codes, I40-41), pre-ECMO cardiac arrest, pre-ECMO chronic renal failure (N18), liver failure pre-ECMO (K70-77), pre-ECMO central nervous system dysfunction (G09-11, G20-21, G23, G31-32, G35-37, G40-41, G45-46, G80-81, S06), and duration of intubation prior to VA-ECMO) [20], hospital volume, IABP, pulmonary artery catheterization, and continuous renal replacement therapy, while also adjusting for within-hospital clustering using a generalized estimating equation [21]. We performed multiple imputation to replace each missing value with a set of substituted plausible values by creating 20 filled-in complete datasets using a Markov chain Monte Carlo algorithm known as “chained equations imputation,” because there were some missing values for BMI and duration of intubation prior to VA-ECMO [22]. Multiple imputation assumes that data are missing at random and that any systematic differences between the missing and observed values can be explained by differences in the observed data [23]. A *p* value <0.05 was considered statistically significant. All statistical analyses were performed using IBM SPSS version 22.0 (IBM Corp., Armonk, NY, USA).

## Results

We identified a total of 5263 patients who received VA-ECMO during the study period. The background characteristics of the patients are presented in Table 1. The largest number of patients was in the cardiogenic shock group (n = 4658). The proportion of male patients in the pulmonary embolism group was smaller than that in the cardiogenic shock group. The mean age ranged from 45.9–68.7 years. In-hospital mortality ranged from 62.0–73.6 %.

**Table 1:** Patient background (n = 5263)

	Cardiogenic shock (n = 4658)		Pulmonary embolism (n = 353)		Hypothermia (n = 99)		Poisoning (n = 50)		Trauma (n = 103)	
Sex										
Male, *n* (%)	3399	(73.0)	131	(37.1)	63	(63.6)	24	(48.0)	73	(70.9)
Age, mean (SD)	64.8	(13.7)	60.6	(15.6)	68.7	(15.2)	45.9	(18.1)	56.4	(20.8)
Death, *n* (%)	3429	(73.6)	226	(64.0)	65	(65.7)	31	(62.0)	66	(64.1)

*SD* standard deviation

Table 2 shows in-hospital mortality and the proportion of patients weaned from VA-ECMO in each of the five etiological categories. Overall, 34.6 % of all patients died during VA-ECMO, 37.9 % died after weaning from VA-ECMO, and 26.5 % were discharged from hospital after weaning from ECMO.

**Table 2:** In-hospital death and weaning from VA-ECMO among patients classified by six etiological categories

	All	Total in-hospital death		In-hospital death under VA-ECMO		Transferred to other hospitals with VA-ECMO		Weaning from VA-ECMO			
								Discharged after weaning from VA-ECMO		In-hospital death after weaning from VA-ECMO	
All, *n* (%)	5263	3817	(72.5)	1823	(34.6)	51	(1.0)	1395	(26.5)	1994	(37.9)
Cardiogenic shock, *n* (%)	4658	3429	(73.6)	1554	(33.4)	44	(0.9)	1185	(25.4)	1875	(40.3)
Pulmonary embolism, *n* (%)*	353	226	(64.0)	151	(42.8)	7	(2.0)	120	(34.0)	75	(21.2)
Hypothermia, *n* (%)*	99	65	(65.7)	49	(49.5)	0	(0.0)	34	(34.3)	16	(16.2)
Poisoning, *n* (%)**	50	31	(62.0)	22	(44.0)	0	(0.0)	19	(38.0)	9	(18.0)
Trauma, *n* (%)*	103	66	(64.1)	47	(45.6)	0	(0.0)	37	(35.9)	19	(18.4)

*p < 0.001 for in-hospital death after weaning from venoarterial extracorporeal membrane oxygenation (VA-ECMO) vs. cardiogenic shock

**p < 0.05 for in-hospital death after weaning from VA-ECMO vs. cardiogenic shock

Table 3 shows in-hospital death and the proportion of patients weaned from VA-ECMO in each of the seven underlying diseases in the cardiogenic shock group. The proportion discharged from hospital after weaning from ECMO was significantly larger in patients with heart failure (31.1 %, *p* < 0.001) and in patients with myocarditis (41.9 %, *p* < 0.001) than in patients with ischemic heart disease (20.3 %). The causes of death after weaning from VA-ECMO in the cardiogenic shock group were: heart failure (n = 580, 30.9 %); infection (n = 169, 9.0 %); hemorrhage (n = 135, 7.2 %); cerebrovascular events (n = 79, 4.2 %); respiratory failure (n = 76, 4.1 %); multiple organ failure (n = 248, 13.2 %); and other (n = 211, 11.3 %). Data on the cause of death were missing for 377 of these patients (20.1). The types of anticoagulant used in the cardiogenic shock group were: heparin (n = 3794, 81.5 %); dalteparin (n = 32, 0.7 %); and argatroban (n = 35, 0.8 %). Data were missing for 797 of these patients (17.1 %). The incidence of hemorrhage was 21.3 % (n = 808) with heparin, 40.6 % (n = 13) with dalteparin, and 22.9 % (n = 8) with argatroban. Two patients also received left ventricular assistance. The numbers of patients who received VA-ECMO within 1, 2, 3–7 and ≥8 days of hospitalization were 2904 (62.3 %), 228 (4.9 %), 589 (12.6 %), and 937 (20.1 %), respectively.

**Table 3:** In-hospital death and weaning from VA-ECMO among patients in the cardiogenic shock group

	All	Total in-hospital death		In-hospital death under VA-ECMO		Transferred to other hospitals with VA-ECMO		Weaning from VA-ECMO			
								Discharged after weaning from VA-ECMO		In-hospital death after weaning from VA-ECMO	
Ischemic heart disease, *n* (%)	1968	1556	(79.1)	725	(36.8)	12	(0.6)	400	(20.3)	831	(42.2)
Heart failure, *n* (%)*	1621	1099	(67.8)	488	(30.1)	18	(1.1)	504	(31.1)	611	(37.7)
Valvular disease, *n* (%)	640	493	(77.0)	246	(38.4)	5	(0.8)	142	(22.2)	247	(38.6)
Myocarditis, *n* (%)*	186	106	(57.0)	24	(12.9)	2	(1.1)	78	(41.9)	82	(44.1)
Cardiomyopathy, *n* (%)	193	141	(73.1)	57	(29.5)	6	(3.1)	46	(23.8)	84	(43.5)
Takotsubo cardiomyopathy, *n* (%)	34	22	(64.7)	8	(23.5)	0	(0.0)	12	(35.3)	14	(41.2)
Infectious endocarditis, *n* (%)	16	12	(75.0)	6	(37.5)	1	(6.3)	3	(18.8)	6	(37.5)

*p < 0.001 for in-hospital death after weaning from venoarterial extracorporeal membrane oxygenation (VA-ECMO) vs. ischemic heart disease

Table 4 shows the in-hospital mortality of patients in the cardiogenic shock group, who had or had not had cardiac arrest, according to the seven underlying cardiac diseases. In-hospital mortality in patients with cardiac arrest ranged from 60.9–100 % and in patients without cardiac arrest it ranged from 54.9 %–77.0 % (*p* < 0.001). In-hospital mortality was significantly lower in patients who had not had cardiac arrest than in those who had had cardiac arrest for those with cardiogenic shock, heart failure and cardiomyopathy (70.5 % vs. 77.1 %, *p* < 0.001; 64.9 % vs. 71.7 %, *p* < 0.05; and 61.0 % vs. 81.0 %, *p* < 0.05, respectively).

**Table 4:** In-hospital mortality among patients in the cardiogenic shock group, who had or had not had cardiac arrest

	Patients who had not had cardiac arrest	Deaths among patients who had not had cardiac arrest		Patients who had had cardiac arrest	Deaths among patients who had had cardiac arrest	
All, *n* (%)*	2471	1742	(70.5)	2187	1687	(77.1)
Ischemic heart disease, *n* (%)	803	618	(77.0)	1165	938	(80.5)
Heart failure, *n* (%)**	922	598	(64.9)	699	501	(71.7)
Valvular disease, *n* (%)	515	391	(75.9)	125	102	(81.6)
Myocarditis, *n* (%)	122	67	(54.9)	64	39	(60.9)
Cardiomyopathy, *n* (%)**	77	47	(61.0)	116	94	(81.0)
Takotsubo cardiomyopathy, *n* (%)	19	12	(63.2)	15	10	(66.7)
Infectious endocarditis, *n* (%)	13	9	(69.2)	3	3	(100.0)

*p < 0.001 for mortality among patients who had not had vs. those who had had cardiac arrest

**p < 0.05 for mortality among patients who had not had vs. those who had had cardiac arrest

Table 5 presents the in-hospital mortality in each category of patients undergoing VA-ECMO for cardiogenic shock. Sex was not significantly associated with mortality. No significant differences in in-hospital mortality were observed between the hospital volume categories.

**Table 5:** In-hospital mortality in patients undergoing VA-ECMO for cardiogenic shock

	Patients, *n*	Deaths, *n* (%)		*P* value
Total	4658	3429	(73.6)	
Age, years				<0.001
19–39	271	170	(62.7)	
40–59	1079	738	(68.4)	
60–79	2741	2058	(75.1)	
≥ 80	567	463	(81.7)	
Sex				0.775
Male	3399	2506	(73.7)	
Female	1259	923	(73.3)	
Hospital volume per year				0.071
0–9	1671	1263	(75.6)	
10–19	1554	1123	(72.3)	
≥ 20	1433	1043	(72.8)	
Body mass index, kg/m^2^				<0.001
< 18.5	406	295	(72.7)	
18.5–24.9	2223	1521	(68.4)	
≥ 25.0	1085	772	(71.2)	
Missing	944	841	(89.1)	
Etiology				<0.001
Ischemic heart disease	1968	1556	(79.1)	
Heart failure	1621	1099	(67.8)	
Valvular disease	640	493	(77.0)	
Myocarditis	186	106	(57.0)	
Cardiomyopathy	193	141	(73.1)	
Takotsubo cardiomyopathy	34	22	(64.7)	
Infectious endocarditis	16	12	(75.0)	
Cardiac arrest				<0.001
No	2471	1742	(70.5)	
Yes	2187	1687	(77.1)	
Chronic renal failure				0.464
No	4139	3040	(73.4)	
Yes	519	389	(75.0)	
Liver failure				0.974
No	4594	3382	(73.6)	
Yes	64	47	(73.4)	
Central nervous system dysfunction				0.527
No	4605	3392	(73.7)	
Yes	53	37	(69.8)	
Duration of intubation prior to VA-ECMO, days				<0.001
0	2576	2007	(77.9)	
1	33	23	(69.7)	
≥ 2	115	66	(57.4)	
Missing	1934	1333	(68.9)	
Use of intra-aortic balloon pumping				<0.001
No	1828	1438	(78.7)	
Yes	2830	1991	(70.4)	
Use of continuous renal replacement therapy				<0.001
No	2893	2058	(71.1)	
Yes	1765	1371	(77.7)	

*VA-ECMO* venoarterial extracorporeal membrane oxygenation

Table 6 shows the results of the multivariable logistic regression analysis for in-hospital mortality, including multiple imputation. Higher age, and greater or smaller BMI were significantly associated with higher mortality. Hospital volume was not significantly associated with mortality.

**Table 6:** Multivariable logistic regression with multiple imputation for analysis of in-hospital mortality in patients undergoing VA-ECMO for cardiogenic shock

	Odds ratio	95 % Confidence interval		*P* value
Age, years				
19–39	Reference			
40–59	1.08	0.79	1.47	0.65
60–79	1.64	1.23	2.17	0.001
≥ 80	2.61	1.81	3.77	<0.001
Sex				
Male	Reference			
Female	0.96	0.82	1.12	0.61
Hospital volume per year				
0–9	Reference			
10–19	0.83	0.68	1.01	0.07
≥ 20	0.80	0.63	1.02	0.07
Body mass index, kg/m^2^				
< 18.5	1.28	1.01	1.62	0.04
18.5–24.9	Reference			
≥ 25.0	1.24	1.04	1.47	0.02
Etiology				
Ischemic heart disease	Reference			
Heart failure	0.55	0.46	0.65	<0.001
Valvular disease	0.85	0.66	1.10	0.22
Myocarditis	0.42	0.31	0.57	<0.001
Cardiomyopathy	0.75	0.51	1.08	0.12
Takotsubo cardiomyopathy	0.58	0.27	1.21	0.14
Infectious endocarditis	0.69	0.21	2.28	0.55
Cardiac arrest				
No	Reference			
Yes	1.52	1.28	1.82	<0.001
Chronic renal failure				
No	Reference			
Yes	0.90	0.69	1.16	0.40
Liver failure pre-ECMO				
No	Reference			
Yes	1.30	0.71	2.38	0.40
Central nervous system dysfunction pre-ECMO				
No	Reference			
Yes	0.87	0.45	1.70	0.68
Duration of intubation prior to VA-ECMO, days				
0	Reference			
1	1.03	0.44	2.39	0.94
≥ 2	0.47	0.32	0.70	<0.001
Use of intra-aortic balloon pumping				
No	Reference			
Yes	0.58	0.49	0.68	<0.001
Use of continuous renal replacement therapy				
No	Reference			
Yes	1.95	1.63	2.33	<0.001

*VA-ECMO* venoarterial extracorporeal membrane oxygenation

## Discussion

The present study investigated the indication for, mortality in, and rate of successful weaning from VA-ECMO, using a Japanese national DPC inpatient database. The majority of patients receiving VA-ECMO had cardiogenic shock (88.5 %). In-hospital mortality was about 65 % for all underlying diseases, and the rate of weaning from VA-ECMO was about 65 %. The advantage of this study was its much larger sample size than was used in previous studies. The present study clarified the practice patterns in the use of VA-ECMO in a nationwide clinical setting. Although there is no evidence on the effectiveness of VA-ECMO for trauma patients, our results showed that VA-ECMO was used for some trauma patients in real-world clinical practice. The present study demonstrated significantly lower mortality in the trauma group compared with the cardiogenic shock group. This may be because patients in the trauma group were relatively younger and had fewer chronic diseases.

In this study we observed the in-hospital mortality under VA-ECMO and weaning from VA-ECMO according to each disease or condition. Mortality during VA-ECMO was 34.6 %, the overall rate of discharge was about 30 %, and in-hospital mortality after weaning from VA-ECMO was about 40 %. About half of patients weaned from VA-ECMO died in hospital. This high proportion may be partly explained by the persisting severe condition of the patients after weaning from VA-ECMO. Furthermore, for some patients weaned from VA-ECMO, further treatment may have been discontinued because of their unfavorable brain condition.

In the cardiogenic shock group, the proportion of in-hospital deaths under VA-ECMO was smaller than that after weaning from VA-ECMO, in contrast with the other groups. This finding may suggest that patients with cardiogenic shock were more likely to have brain damage than other groups of patients, resulting in a lower survival rate. Only two patients received implantation of a left ventricular assistance device. Implantation of a left ventricular assistance device has only been approved as a bridge to transplantation since 2011. However, switching from VA-ECMO to a left ventricular assistance device is not popular, because of the severe shortage of cardiac donors in Japan [24].

In-hospital mortality was lower in patients who had not had cardiac arrest than in those who had had cardiac arrest. This finding indicates that cardiac arrest may also be a predictor of VA-ECMO outcome.

In-hospital mortality was higher in older patients and in patients with high or low BMI. This indicates that successful outcomes for VA-ECMO in the cardiogenic group were associated with younger age and appropriate BMI. This finding could suggest that age and BMI are predictors of VA-ECMO outcome. Hospital volume was not significantly associated with in-hospital mortality. High-volume hospitals may treat severely ill patients on VA-ECMO more than low-volume hospitals because of differences in the criteria for implementing VA-ECMO. Use of IABP was associated with lower mortality. It is recognized that VA-ECMO increases cardiac afterload due to its reversed vascular flow. Consequently, VA-ECMO reduces cardiac output and causes left ventricular distension [25]. As IABP reduces afterload and increases coronary blood flow [26], it may have reduced mortality by increasing cardiac output and decompressing the left ventricle.

There are some limitations in this study. This study was retrospective and was based on data from an administrative database, which did not include complete data on physiology, severity of illness, the patient’s physical condition, or duration of time between cardiac arrest and initiation of VA-ECMO. Generally, in retrospective observational studies using administrative data, recorded diagnoses are less well-validated than those in planned prospective surveys. Severe organ dysfunction may have been more likely to be recorded than mild-to-moderate dysfunction. The lack of a control group precluded any conclusion as to whether there was an association between use of VA-ECMO and mortality. Because of the way that data were recorded, we were also not able to differentiate patients who were in cardiac arrest before ECMO was initiated from those in whom ECMO was established for ongoing cardiac arrest during cardiopulmonary resuscitation.

## Conclusions

This study was based on data from a nationwide database in Japan to determine mortality rates in patients who received VA-ECMO. Mortality rates were high, especially in patients with cardiogenic shock and in patients who had cardiac arrest. Weaning from VA-ECMO did not necessarily result in survival. The present study provides information about the current status of VA-ECMO use in Japan; further studies are needed to investigate the effects of VA-ECMO.

## Key messages

- In-hospital mortality was 72.5 % in 5263 patients receiving VA-ECMO
- More than 50 % of patients weaned from VA-ECMO died in hospital
- Age, BMI and cardiac arrest were predictors of mortality after VA-ECMO

## Abbreviations

BMI: body mass index
DPC: Diagnosis Procedure Combination
IABP: intra-aortic balloon pumping
ICD-10: *International Classification of Diseases 10th Revision*
VA-ECMO: venoarterial extracorporeal membrane oxygenation
